# High-Throughput Voigt Deconvolution of Luminescence Spectral Maps with Curvature-Informed Peak Initialization

**DOI:** 10.1021/acsomega.6c07689

**Published:** 2026-10-21

**Authors:** Melissa A. Wunch, Luna Garcia, Jordan Barry, Chris Thron, Taylor B. Harvey

**Affiliations:** Department of Science and Mathematics, 143061 Texas A&M University-Central Texas, Killeen, Texas 76549, United States

## Abstract

Accurate deconvolution of overlapping emission bands remains a key challenge in luminescence spectroscopy, particularly for large-scale data sets generated by cathodoluminescence (CL) and photoluminescence (PL) mapping. This work presents a scalable Python-based workflow for high-throughput Voigt deconvolution of CL spectra. The approach integrates curvature-informed peak initialization with nonlinear least-squares optimization to improve fit stability and consistency across large data sets and extract quantitative spectral parameters, including emission energy, amplitude, and Gaussian and Lorentzian broadening contributions. The resulting parameters enable direct statistical analysis and spatial mapping across data sets. Synthetic validation and full-map uncertainty analysis show that peak center and total line width are more robustly recovered than the individual Gaussian and Lorentzian contributions when spectral features strongly overlap. Application to CdMgSeTe thin films demonstrates spatial variation in emission behavior and distinguishes differences in spectral heterogeneity between the analyzed films. This workflow enables high-throughput conversion of hyperspectral CL maps into spatially resolved spectral fingerprints with peak-level detail suitable for quantitative comparison across samples and processing conditions.

## Graphical abstract

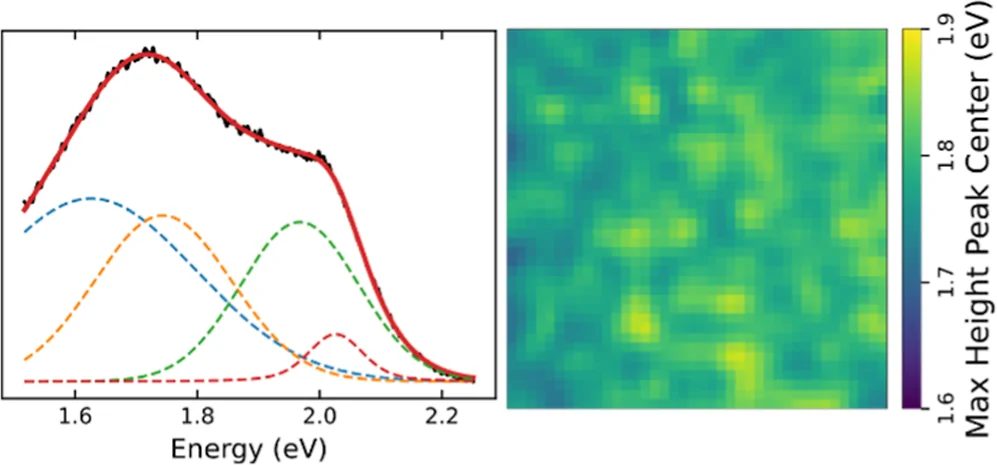

## 1 Introduction

Luminescence spectroscopy is a powerful technique for probing the optical and electronic properties of materials, offering high sensitivity to variations in composition, structure, and defect states.^1–4^ As spectroscopic instrumentation continues to advance, there has been an increase in the reliance on spatially resolved techniques to analyze complex materials at the microscale and nanoscale. These techniques include cathodoluminescence (CL) and photoluminescence (PL) mapping.^1,5–8^ The hyperspectral data sets generated can reveal detailed insights into heterogeneities and functional behavior, but they also present significant challenges in data interpretation.^9,10^

One of the central obstacles in luminescence spectral analysis is the frequent occurrence of overlapping emission bands, which often result from a combination of physical processes. These can include defect recombination, impurity states or localized states, structural disorder, and phonon interactions.^11–13^ Simple fitting models, such as individual Gaussians, often fail to resolve such features accurately, particularly when applied across large data sets. Manual or semiautomated fitting approaches are also impractical for modern high-throughput analysis, leading to a pressing need for automated, scalable, and physically grounded deconvolution tools.^14^

The Voigt profile, which uses a convolution of Gaussian and Lorentzian functions, offers a more complete representation of spectral line shapes than either component alone.^15^ It was introduced to describe absorption and emission lines in atomic and molecular spectroscopy, but has since become a standard model across Raman, infrared, and X-ray spectroscopy.^16–18^ In the context of luminescence, the Gaussian and Lorentzian contributions can provide information about different broadening behaviors. Gaussian-like broadening is commonly associated with instrumental resolution and static disorder, while Lorentzian-like broadening may reflect homogeneous processes such as exciton lifetimes, collision-induced effects, and recombination kinetics.^19^ The Voigt function’s ability to characterize these contributions enables deeper physical interpretation of the properties of these materials.

Despite its utility, the Voigt profile does pose some challenges. It does not have a closed-form analytical solution and must be evaluated using special functions such as the Faddeeva function or via pseudo-Voigt approximations.^5,15^ Fitting overlapping Voigt components to complex, high-dimensional data, especially in hyperspectral maps, requires robust optimization strategies, careful parameter initialization, and noise mitigation.^20–22^ These factors have historically limited the widespread use of Voigt fitting in large-scale luminescence data sets.

In response to this need, this work presents a Python-based workflow for high-throughput Voigt deconvolution of luminescence spectral maps. The approach combines curvature-informed peak initialization, structured line width optimization, constrained model-order selection, and nonlinear least-squares fitting to resolve overlapping emission bands across large cathodoluminescence data sets. Rather than relying on manual spectrum-by-spectrum fitting, the workflow applies a consistent fitting framework across full spectral maps, enabling reproducible extraction of peak-resolved parameters, including center energy, amplitude, Gaussian width (σ), Lorentzian width (γ), peak height, and peak area.

Application to CdMgSeTe thin films demonstrates how the method converts hyperspectral CL data into spatially resolved and statistically interpretable descriptors of emission behavior. The fitted parameters distinguish clear differences between the two films in spatial variability and spectral complexity. These results show that high-throughput Voigt deconvolution can extend CL analysis beyond conventional intensity or peak-position mapping by generating quantitative spectral fingerprints that capture both local heterogeneity and population-level trends. Applying the workflow across full spectral maps also enables systematic evaluation of where fitted parameters remain robust and where spectral overlap limits unique interpretation, revealing population-level fitting behavior that would be difficult to establish from a small number of representative deconvolutions alone. Building on previous cathodoluminescence mapping work,^23^ this framework provides a scalable and reproducible pathway for analyzing complex semiconductor emission behavior across large experimental data sets.

## 2 Materials and Methods

### 2.1 Thin Film Synthesis and Characterization

The process of thin film synthesis and scanning electron microscope (SEM) and cathodoluminescence (CL) acquisition parameters used for this work has been reported previously.^23^ In brief, CL mapping was acquired for a CdMgSeTe thin film with a Mg sublimation temperature of 435 °C (sample Mg-435) and 450 °C (sample Mg-450) using a JEOL 7610F SEM equipped with a Horiba iHR-320 CL detector. CL measurements were obtained with an accelerating voltage of 5–10 kV and a probe current of 13 μA and captured using a CCD Syncerity detector in the 500–900 nm spectral range. An SEM image of an example acquisition map is shown in Figure S1.

### 2.2 Python Algorithm

A Python-based algorithm was developed to perform automated Voigt deconvolution of cathodoluminescence (CL) spectra using a structured, multistage workflow. The implementation was executed in Python 3.13 (Spyder IDE) and is designed for batch processing of hyperspectral data sets. The complete curvature-initialized Voigt fitting algorithm is provided as pseudocode in the Supporting Information.

#### 2.2.1 Spectral Preprocessing

Spectral data were acquired as intensity versus wavelength and converted to an energy basis using a Jacobian correction to preserve spectral density.^24^ Following transformation, spectra were interpolated onto a uniform energy grid to ensure numerical stability during fitting.

Two smoothing strategies were applied. Fourier-based smoothing was used for derivative-based peak detection (Section 2.2.2), and Gaussian smoothing for final nonlinear fitting (Section 2.2). Fourier smoothing with 50 endpoint points and 10 retained components was used for derivative-based peak identification. Final data for nonlinear fitting were Gaussian-smoothed with σ = 2 grid points, meaning the Gaussian kernel standard deviation was set to two sampling intervals on the uniformly interpolated energy axis. This separation reduced high-frequency noise during candidate detection while retaining a consistent, lightly smoothed signal for parameter estimation.

No additional baseline subtraction was performed, as baseline correction was applied during acquisition.

#### 2.2.2 Peak Candidate Identification

Initial peak locations were estimated from the Fourier smoothed data using a curvature-based approach described previously.^25^ Local minima in the second derivative of the smoothed spectrum were identified as candidate peak centers. To improve robustness, the candidate peak centers were then filtered using thresholding based on a fraction of a robust curvature scale (95th percentile) and minimum separation constraint between peaks (expressed as a fraction of total energy range). Representative values used in this work were a threshold fraction of 0.10 and a minimum separation of 0.10 (fraction of energy span).

This process yields a reduced set of well-separated candidate peaks used for model initialization.

#### 2.2.3 Model Definition and Initialization

Luminescence spectra deconvolution is performed by fitting the measured signal *S*(*x*) as a sum of Voigt-shaped peaks

1 $$S\left(x\right)=\sum A_{i}V\left(x-x_{i},\sigma_{i},\gamma_{i}\right)+B\left(x\right)$$

where *A*_*i*_, *x*_*i*_, σ_*i*_, γ_*i*_ are the fitted intensity, center position, Gaussian broadening, and Lorentzian broadening, respectively, of the *i*-th peak; *V*(*x* – *x*_*i*_, σ_*i*_, γ_*i*_) denotes the Voigt function evaluated via the Faddeeva formulation; and *B*(*x*) represents the background signal. For this analysis, baseline subtraction was already performed by the CL acquisition equipment and *B*(*x*) = 0.

Initial parameter values were assigned for peak centers from curvature-based detection and amplitudes and widths from physically reasonable estimates with bounded constraints. The Voigt function was implemented using the Faddeeva formulation.^15,19^

#### 2.2.4 Nonlinear Least-Squares Fitting

Parameter optimization was performed using nonlinear least-squares fitting (SciPy). All peak parameters were optimized simultaneously.

Parameter bounds were applied to maintain physically reasonable solutions and improve numerical stability. Peak amplitudes were constrained to nonnegative values, while peak centers were permitted to vary within ±10% of the measured energy span relative to their initialized positions, subject to the limits of the acquired spectral range. γ and σ were assigned a small positive numerical lower bound of 2Δ*E*, where Δ*E* is the uniform energy-grid spacing. For γ, this served as a physically motivated regularization to prevent convergence to the pure-Gaussian limit in broad semiconductor emission features where both homogeneous and inhomogeneous broadening contributions are expected. Upper bounds were defined relative to the measured energy span: γ was limited to 12.5% of the span and σ to 25% for fully contained peaks.

To improve sensitivity to peak structure, a curvature-weighted objective function was employed, increasing the influence of high-curvature (feature-rich) regions relative to flat background regions. To emphasize regions containing stronger spectral structure, the nonlinear least-squares fit used curvature-based relative weighting. Pointwise weights were defined from the magnitude of the second derivative and normalized by the 95th percentile curvature magnitude, with a weighting factor of 3. These weights were used only to prioritize high-curvature regions during optimization and should not be interpreted as experimental measurement uncertainties. A full description of the weighting is given in the Supporting Information.

#### 2.2.5 Line Width Optimization Strategy

To address sensitivity to initial line width parameters, a structured parameter sweep over Gaussian (σ) and Lorentzian (γ) initial values was performed. A 5 × 5 logarithmically spaced grid of representative initial σ and γ starting values was evaluated for each spectrum. The tested initial values spanned approximately 2.0–12.6% of the fitted energy range for σ and 1.0–6.3% for γ. For each initialization combination the nonlinear fit was executed, the sum of squared errors (SSE) was computed, the lowest-SSE solution was retained and used to seed the final bounded nonlinear fit, in which the line width parameters were free to vary over the full allowed ranges.

#### 2.2.6 Model Order Selection

The number of Voigt components (*n*) was determined using a constrained, multistep approach. Initial candidate counts were derived from curvature-based analysis of each spectrum, providing a data-informed range of plausible peak numbers. From this range, a fixed model order was selected to represent the dominant candidate behavior. The selected model order was then evaluated using several complementary model selection metrics, including the residual sum of squares (RSS), Akaike information criterion (AIC), Bayesian information criterion (BIC), and penalized RSS (PRSS). These metrics were used as supporting diagnostics rather than as the sole basis for model-order selection. Based on this combined assessment, a single model order was applied consistently across each data set to enable direct comparative analysis while reducing computational cost.

#### 2.2.7 Batch Processing and Output

Once the order number for a CL data set was chosen, the algorithm was applied to full hyperspectral data sets, enabling automated processing of all spectra. Full-data set analyses were performed in Python 3.13.9 using SciPy 1.16.3 on a Windows 11 system equipped with an Intel Core Ultra 7 255H processor and 32 GB RAM. Reported runtimes include the two-dimensional σ–γ initialization grid search applied to each spectrum. For each fitted peak, the peak center energy, amplitude, Gaussian width (σ), Lorentzian width (γ), and derived quantities (peak height and area) were extracted. The total Voigt full width at half-maximum (fwhm) was calculated from the fitted Gaussian and Lorentzian line width terms using the Olivero–Longbothum approximation.

2 $${FWHM}_{V}\approx 0.5346\left(2\gamma \right)+{\left[0.2166{\left(2\gamma \right)}^{2}+{\left(2.35482\sigma \right)}^{2}\right]}^{1/2}$$

Outputs were stored in structured formats for downstream statistical analysis and spatial mapping.

#### 2.2.8 Synthetic Validation

A synthetic validation study was conducted using spectra generated from known Voigt components and processed with the same fitting workflow applied to the experimental CL data. Two cases were evaluated: a well-resolved mixed-Voigt spectrum and a data-like spectrum containing broader, overlapping lower-energy components with a more exposed high-energy peak. Fifty independent realizations were generated for each case using 1.5% point-to-point Gaussian noise relative to the spectrum maximum and 5% spectrum-to-spectrum intensity jitter. Recovery of peak centers, Gaussian and Lorentzian line width terms, and total Voigt fwhm was then compared with the known input values.

## 3 Results and Discussion

To address the need for accurate and scalable analysis of complex luminescence spectra, a Python-based algorithm for automated Voigt profile deconvolution was developed. This approach employs Voigt line shape models to resolve overlapping emission bands and extract peak-resolved parameters from spectroscopic data.^13,20^ The algorithm was initially developed for individual spectra and subsequently extended to batch processing of large spatial maps, enabling rapid and reproducible analysis. Such approaches have proven effective in hyperspectral imaging of thin films,^26,27^ providing insight into material inhomogeneities and local electronic environments. In the present study, this framework is applied to cathodoluminescence (CL) maps of CdMgSeTe thin films synthesized under different Mg sublimation conditions (Mg-435 and Mg-450), enabling direct comparison of spectral structure and spatial variability between the two films.

Figure 1 illustrates the output of the preprocessing steps applied prior to fitting on a representative spectrum from Mg-450. The raw spectra, originally acquired in wavelength space, were converted to energy using a Jacobian correction and interpolated onto a uniform energy grid. This transformation preserves the overall spectral structure while enabling physically consistent peak modeling. Gaussian smoothing was then applied to reduce high-frequency noise while maintaining peak shape, producing the final spectra used for Voigt fitting.

**Figure 1:**
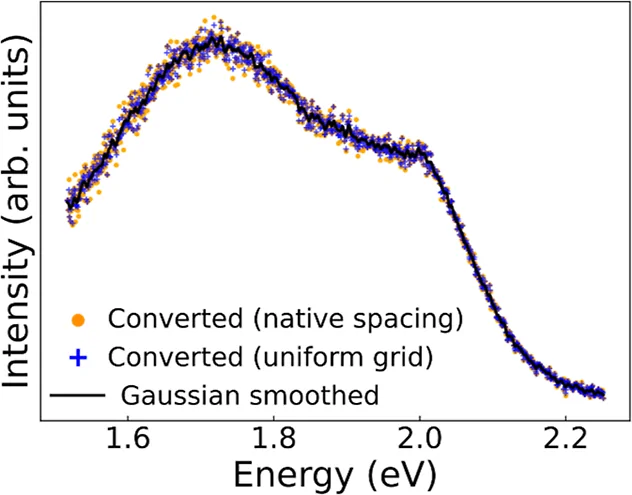
Measured data converted to energy before (orange dot) and after (blue plus) interpolation and the Gaussian-smoothed data (black line) for an example spectrum.

Decomposition into multiple Voigt components introduces a highly nonconvex optimization problem, making the fit sensitive to initial parameter estimates, particularly peak centers. To address this, initial peak locations were determined using a curvature-based approach described previously,^25^ where local minima in the second derivative of the spectrum were used as candidate peaks. Prior to differentiation, Fourier-based smoothing was applied to suppress high-frequency noise and stabilize the derivative, enabling robust identification of physically meaningful peak centers.

A sequence of filtering criteria was applied to the curvature-derived candidates to remove spurious detections. Candidates were screened based on their relative magnitude using a robust curvature scale, and a minimum separation constraint was imposed to prevent multiple detections within a single broadened feature. As shown in Figure 2, raw second-derivative minima systematically overestimate peak count, while application of these constraints produces a reduced and more consistent set of well-separated candidate peaks.

**Figure 2:**
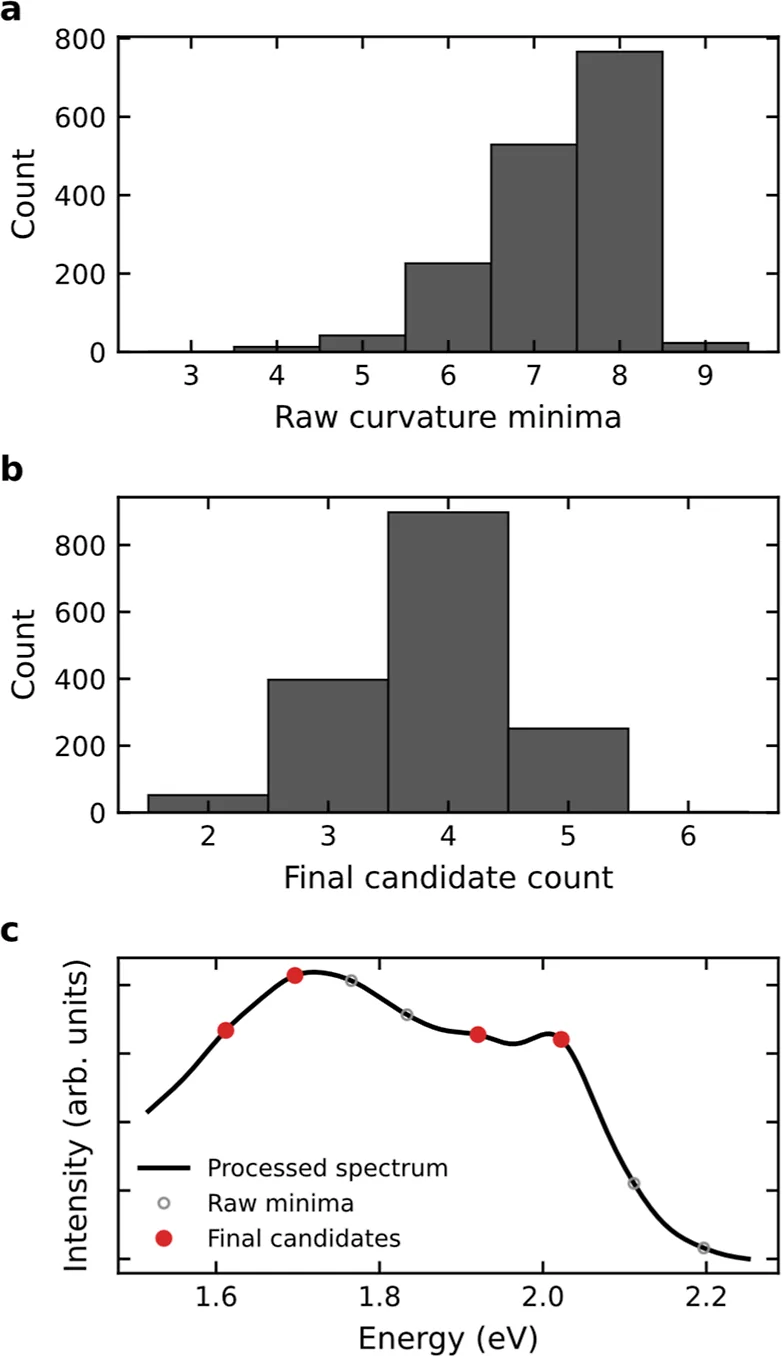
Curvature-based peak candidate refinement. Raw second-derivative minima (a) systematically overestimate peak count, while application of scale-based thresholding and minimum separation constraints (b) yields a reduced and more stable candidate set. A representative spectrum (c) illustrates the progression from initial curvature minima to final peak candidates.

Table 1 shows the candidate peak distribution for the two films in this study. The candidate-count distributions provide an initial basis for selecting a fixed model complexity for each data set prior to full Voigt analysis. For Mg-435, the filtered candidate counts were concentrated at two and three peaks, with three peaks selected as the working model order. Although both values occurred with similar frequency, the higher count was chosen to provide sufficient model flexibility to capture partially overlapping or unresolved spectral features that may not be consistently identified across all spectra. This choice reflects a controlled preference for avoiding underfitting while remaining within the dominant candidate range defined by the curvature-based filtering.

**Table 1:** Distribution of Final Candidate Peak Counts after Curvature-Based Thresholding and Minimum Spacing Constraints for the Mg-435 and Mg-450 Datasets^a^

# peaks	Mg-435 (*n*, %)	Mg-450 (*n*, %)
1	22 (1.4%)	—
2	780 (48.8%)	52 (3.2%)
3	770 (48.1%)	397 (24.8%)
4	28 (1.8%)	898 (56.2%)
5	—	251 (15.7%)
6	—	2 (0.1%)

a Values are reported as the number of spectra with the corresponding candidate count, with percentages in parentheses indicating the fraction of the total dataset.

For Mg-450, the distribution was more strongly centered at four peaks, which represented the most common candidate count by a clear margin. Accordingly, four curves were selected as the initial model order, as this choice captures the predominant spectral complexity while avoiding the additional variability introduced by less frequent higher-order candidates.

In both cases, the selected model orders intentionally favor slightly higher complexity within the constrained candidate range, allowing the fitting procedure to better accommodate subtle spectral structure. To further evaluate the adequacy of these selections, the identified candidate peaks were used to initialize a nonlinear fitting framework based on a sum of Voigt functions.

Following identification of candidate peak locations, each spectrum was modeled as a superposition of Voigt components and fit using nonlinear least-squares optimization. Gaussian smoothing was applied prior to fitting, rather than the Fourier smoothing used for peak detection, as it better preserves peak shape while reducing noise in the fitting stage. Candidate peak positions were used to initialize the center parameters, while amplitudes and line widths were assigned physically reasonable initial values and bounds. All peak parameters, including amplitude, center, Gaussian width (σ), and Lorentzian width (γ), were then simultaneously optimized. This approach combines curvature-based peak detection for informed initialization with full-spectrum fitting to refine parameter estimates.

Because Voigt fitting is sensitive to line width initialization, a parameter sweep over Gaussian (σ) and Lorentzian (γ) starting values was performed, with the lowest-SSE solution retained. Figure 3 shows a representative example from the Mg-450 data set. The one-dimensional sweep (Figure 3c) demonstrates a clear dependence of fit quality on σ, with a well-defined minimum indicating an optimal line width range. However, the two-dimensional σ–γ grid (Figure 3d) reveals a broader region of comparably low SSE values surrounding this minimum, indicating that multiple parameter combinations yield similarly acceptable fits. This behavior highlights that, while a best-fit solution can be identified, the decomposition is not uniquely defined by a single initialization and instead reflects a stable solution space across plausible line width values.

**Figure 3:**
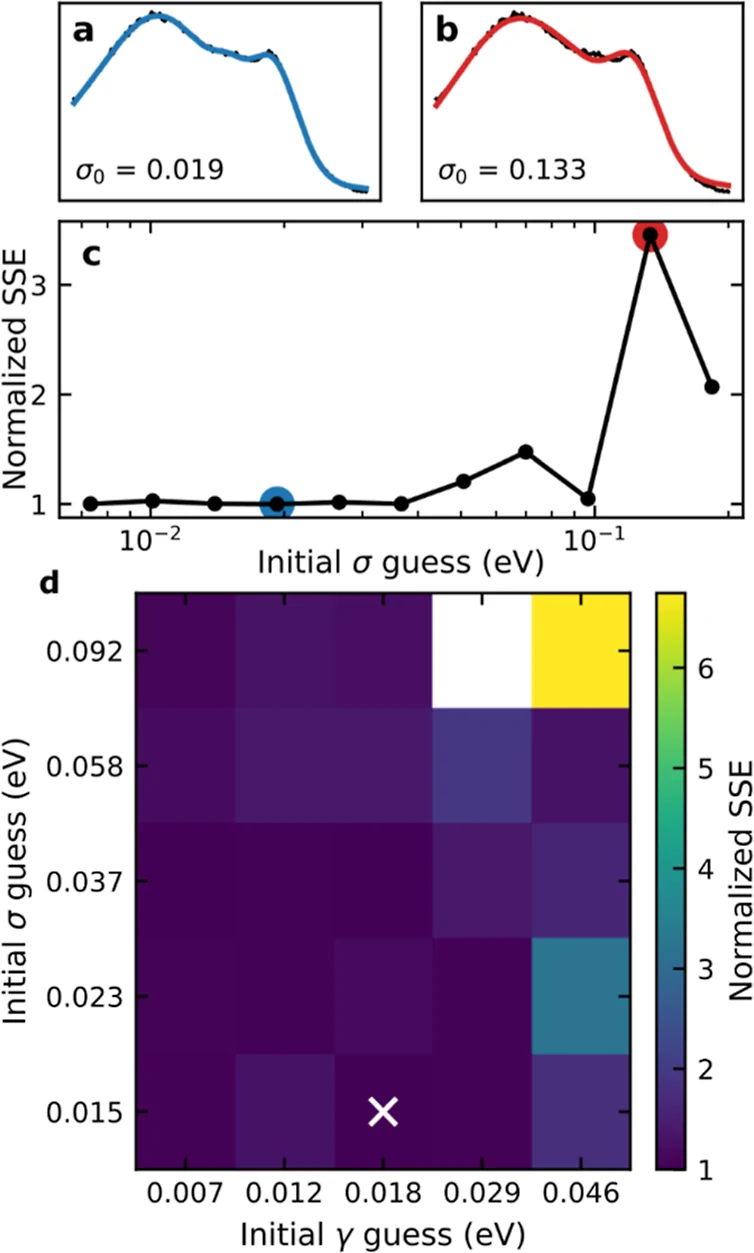
Evaluation of Voigt line width initialization through structured parameter sweeps. One-dimensional sweep of σ with γ held constant, illustrating sensitivity of fit quality to Gaussian broadening for (a) fit with lowest sum of squared errors (SSE), (b) fit with highest SSE and the (c) SSE as a function of Gaussian broadening. (d) Two-dimensional grid search over Gaussian (σ) and Lorentzian (γ) initial values, with the SSE shown for each combination; the minimum SSE solution is indicated by the white × and a white box indicates where the solver did not converge.

To assess the selected model order, the number of Voigt components (*n*) was systematically varied and the resulting fits were evaluated. Figure 4 shows a representative example from the Mg-450 data set. As expected, the residual sum of squares (RSS) decreases with increasing *n*; however, the penalized metric (PRSS) exhibits a clear minimum, indicating a balance between fit quality and model complexity. For the representative spectra evaluated, these metrics indicated that improvements in fit quality became marginal beyond a limited range of *n*, supporting the use of a constrained model order derived from the data set-wide curvature-based candidate analysis.^28,29^ This behavior was also observed in full-data set comparisons using alternative fixed model orders, where additional components generally reduced residual error but redistributed overlapping spectral contributions, reinforcing the need to constrain model order using the data set-wide candidate distribution rather than fit error alone. Figure S2 shows additional metrics employed in evaluation of the number of Voigt components.

**Figure 4:**
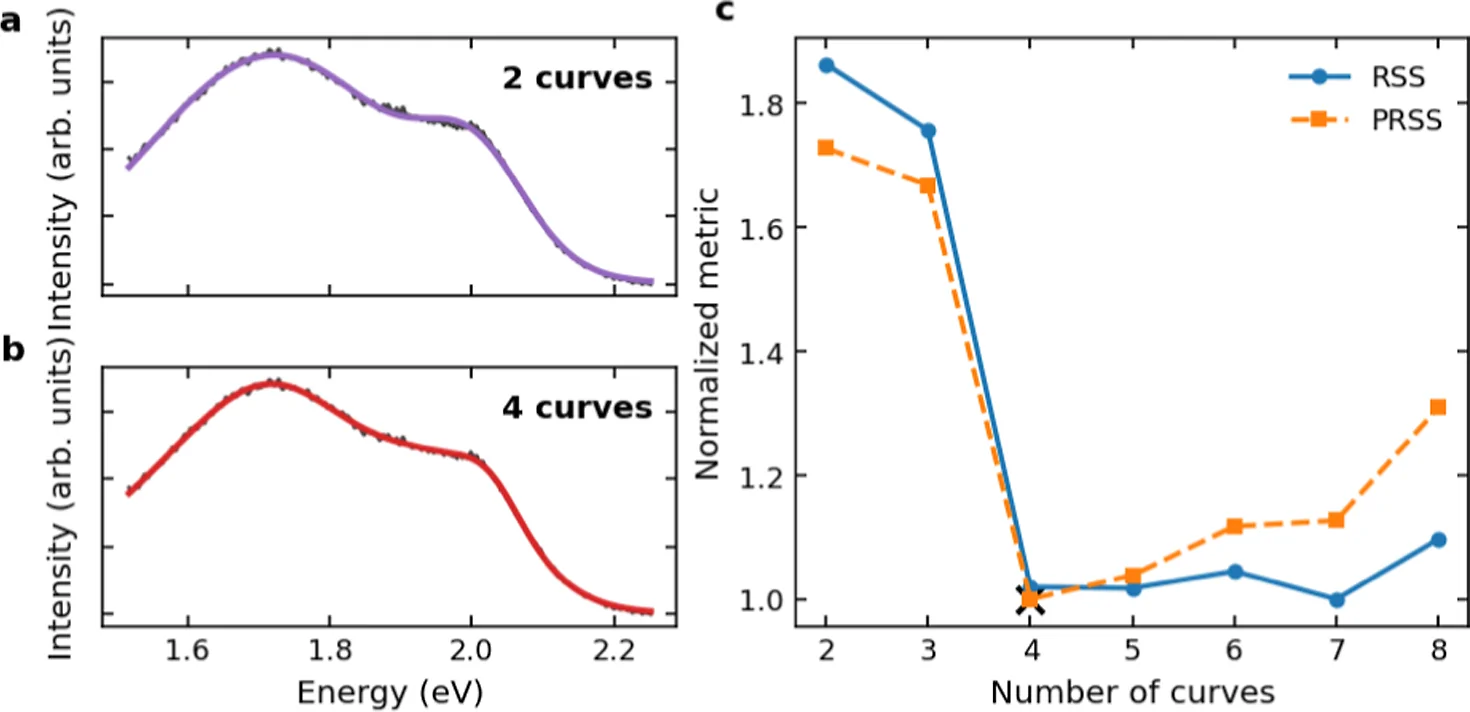
Representative curve-count sweep analysis for a single spectrum. Panels (a) and (b) compare the Voigt fits obtained using the minimum (2 curves) and selected number of curves, respectively, overlaid on the preprocessed data. Panel (c) shows the model selection behavior as a function of curve count using normalized residual sum of squares (RSS) and penalized RSS (PRSS). While RSS decreases monotonically with increasing model complexity, PRSS introduces a penalty for additional peaks and exhibits a minimum at the selected model (marked with ×), balancing fit quality and model parsimony.

Although the fitting procedure provides a quantitative decomposition, the number of peaks should not be selected based solely on statistical improvement. Each component may represent a physical emission process, and increasing model complexity will generally reduce fitting error but may introduce nonphysical features. Accordingly, the model order was constrained using curvature-based candidate selection and evaluated using model selection metrics to ensure a balance between fit quality and physical interpretability. While formal regularization approaches (e.g., LASSO) could further automate model selection, they introduce additional assumptions and were not considered here.^30^

Once the number of peaks is selected, the fitting procedure enables extraction of peak parameters, including peak center, amplitude, and broadening components (σ and γ). Figure 5 illustrates representative Voigt deconvolutions of CL spectra using the described multipeak fitting framework for Mg-435 (Figure 5a) and Mg-450 (Figure 5b). In both examples, the fitted components collectively reproduce the measured signal while resolving distinct spectral contributions that are not readily separable in the raw spectra. The decomposition captures both narrow spectral features and broader underlying contributions while maintaining consistency with the overall spectral profile. The representative spectra were both reproduced with high overall fit quality. Mg-435 spectrum 11 yielded a coefficient of determination, *R* ^2^, of 0.9968 and a normalized root-mean-square error (NRMSE) of 1.62%, while Mg-450 spectrum 752 yielded *R* ^2^ = 0.9993 and an NRMSE of 0.85%. These values indicate close agreement between the measured spectra and the summed fitted profiles.

**Figure 5:**
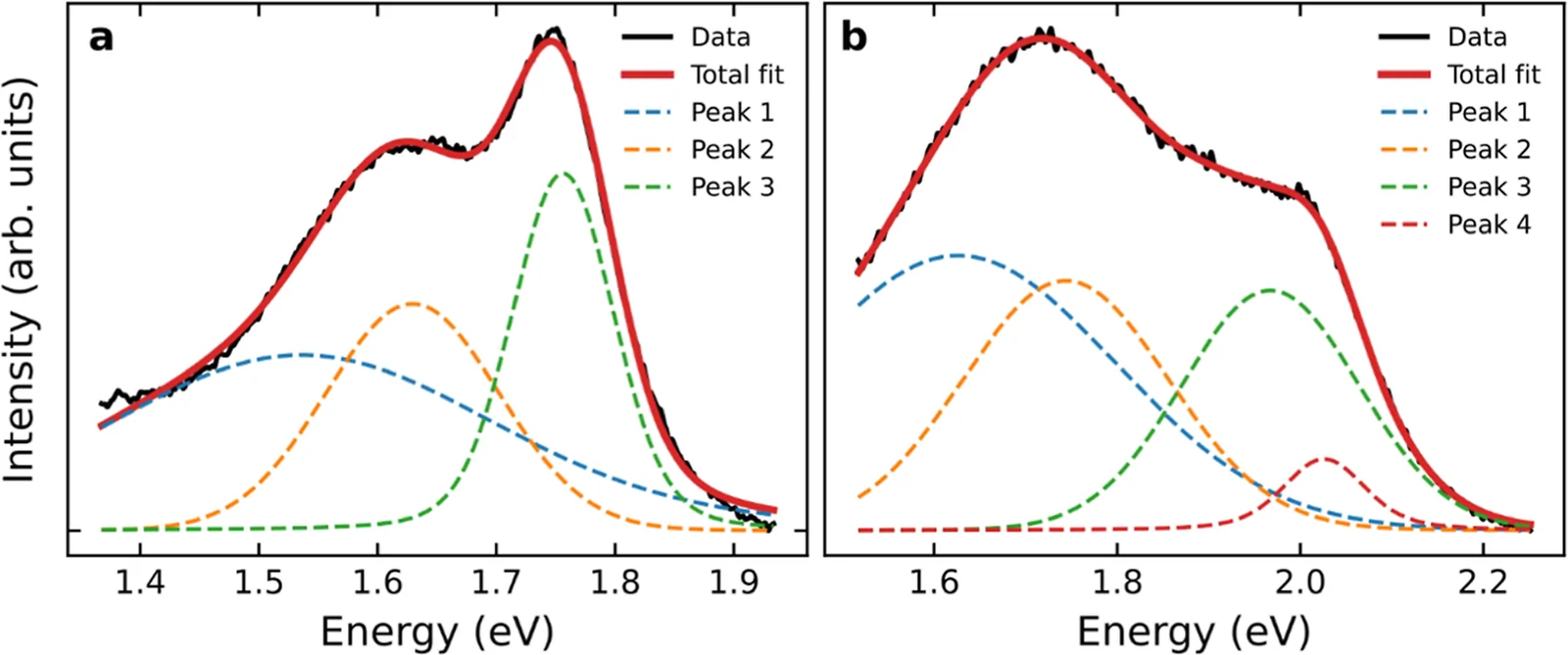
Voigt deconvolution of a single CL measurement for (a) Mg-435 and (b) Mg-450. Experimental data are shown in black, the cumulative Voigt fit in red and the individual Voigt peaks as dotted lines colored blue, orange, green, red in order of ascending center point.

Cathodoluminescence (CL) spectra were acquired with instrument-level baseline correction; therefore, no additional baseline subtraction was applied. In CL mapping, constrained acquisition ranges can truncate low-intensity shoulders or extended emission tails. Additional baseline correction can further suppress these features, potentially removing physically meaningful contributions. By retaining the full spectral range without further modification, these broader and lower-intensity features are preserved in the fitting, as observed for both samples in Figure 5.

The fitted parameters for the representative spectra are summarized in Table 2 for both films in Figure 5. For Mg-435 spectrum 11, the highest peak intensity arises from peak 3 despite it not having the largest amplitude, reflecting its relatively narrow width and efficient concentration of intensity. For Mg-450 spectrum 752, peak 4 is clearly resolved but contributes less to the overall intensity, consistent with its lower amplitude and area. In both cases, the lower-energy components are broader and more strongly overlapping, particularly in the Mg-450 spectrum, where they collectively describe the extended low-energy emission. A distinct Lorentzian contribution is most consistently recovered in the highest-energy peak. The lower-energy components generally recover smaller Lorentzian terms, although the subsequent uncertainty analysis shows that the individual line width contributions are not independently well constrained for these overlapping features. The uncertainty and identifiability of these fitted line width terms are considered further below.

**Table 2:** Fitted Parameters of Representative Spectra from Mg-435 and Mg-450, Including Center Energy, Amplitude, Gaussian Width (σ), Lorentzian Width (γ), Total Voigt Full Width at Half Maximum (FWHM), Peak Height, and Peak Area

spectra	peak no.	peak center (eV)	amplitude (arb. units eV)	σ (eV)	γ (eV)	Voigt fwhm (eV)	peak height (arb. units)	peak area (arb. units eV)
Mg-435 spectrum 11	1	1.536	1.89 × 10^5^	0.141	0.049	0.389	4.12 × 10^5^	1.44 × 10^5^
	2	1.629	9.79 × 10^4^	0.072	0.001	0.172	5.33 × 10^5^	9.77 × 10^4^
	3	1.756	9.74 × 10^4^	0.039	0.009	0.101	8.39 × 10^5^	9.50 × 10^4^
Mg-450 spectrum 752	1	1.626	1.65 × 10^5^	0.171	0.002	0.406	3.81 × 10^5^	1.21 × 10^5^
	2	1.744	9.82 × 10^4^	0.111	0.003	0.264	3.46 × 10^5^	9.55 × 10^4^
	3	1.967	8.13 × 10^4^	0.096	0.002	0.228	3.33 × 10^5^	8.09 × 10^4^
	4	2.026	1.31 × 10^4^	0.035	0.021	0.106	9.90 × 10^4^	1.25 × 10^4^

To assess how reliably the fitting workflow recovers known spectral parameters under controlled conditions, a synthetic validation study was performed using spectra generated from known Voigt components. Two representative cases were evaluated: a well-resolved spectrum with four distinct mixed-Voigt components and a data-like spectrum containing broader, more strongly overlapping low-energy contributions together with a more exposed high-energy peak. These scenarios were selected to bracket the range of spectral separation encountered in the experimental data while keeping the validation focused on conditions most relevant to the present study. Synthetic validation showed accurate recovery of peak position and total Voigt line width for the well-resolved case and for the more spectrally isolated components of the data-like overlapping case. As shown in Figure 6a,b, the recovered component sums closely reproduce the known synthetic spectra in both scenarios, with *R* ^2^ values greater than 0.999 and NRMSE below 0.7%. The Supporting Information contains detailed recovery statistics on a parameter basis that is briefly described here. Figure S3a shows that peak-center recovery is highly accurate for the well-resolved case and improves across the data-like spectrum toward the more isolated high-energy component. Figure S3b compares the mean absolute relative errors in σ, γ, and total Voigt fwhm, showing that fwhm is recovered more consistently than the individual line width terms, while γ exhibits the largest errors. Recovery degraded for the broad, overlapping low-energy components, where neighboring features obscured the shoulders and tails needed for unique parameter separation. In contrast, the exposed highest-energy component was recovered most consistently, including both its center position and total line width.

**Figure 6:**
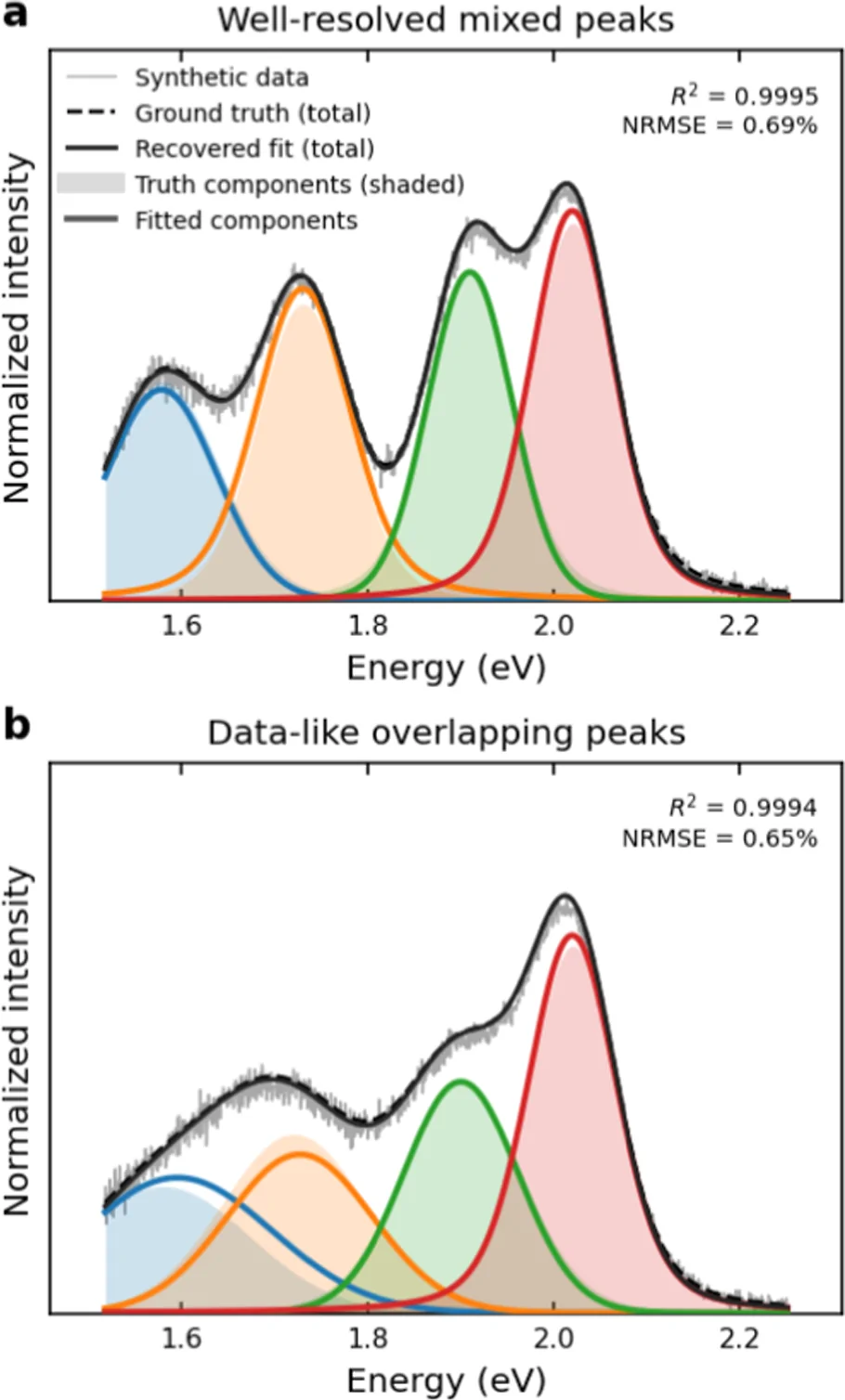
Representative synthetic validation spectra for (a) well-resolved mixed peaks and (b) data-like overlapping peaks. Shaded curves indicate the known component contributions, colored lines show the recovered fitted components, and the total recovered fit is compared with the ground-truth spectrum. Both cases show close agreement between the recovered and known spectra.

The synthetic validation therefore emphasizes that parameter recoverability depends strongly on spectral overlap and the amount of exposed line-shape information available to the fit. The highest-energy component provides the clearest example of this behavior, while the broader low-energy components illustrate the limitations imposed by overlapping shoulders and tails. The synthetic study was intentionally limited to two representative scenarios most relevant to the experimental data. A broader identifiability analysis could systematically examine the effects of spectral overlap, noise, resolution, and model constraints; such an analysis is beyond the scope of the present work, but the current validation provides a practical basis for interpreting the experimental fits and for evaluating parameter uncertainty across the full spectral maps.

The full data set was then analyzed using a consistent model order and identical fitting procedure across each spectral map, enabling direct comparison of parameters across the map. Because the full-map results encompass the range of spectral conditions encountered experimentally, they provide the appropriate basis for assessing overall fitting performance, computational efficiency, and parameter uncertainty.

Across the full spectral maps, the fitting workflow converged successfully for all 1600 spectra in both data sets. Mg-435 was fit using three components and required approximately 3699 s of wall time, or 2.31 s per spectrum, while Mg-450 used four components and required approximately 7864 s, or 4.92 s per spectrum. Fit quality remained consistently high across both data sets, with median *R* ^2^ values of 0.9967 and 0.9989 and median NRMSE of 1.64% and 1.02% for Mg-435 and Mg-450, respectively. The 100% convergence rate and consistently low fitting error demonstrate that the workflow can be applied reproducibly across full hyperspectral maps.

Covariance-derived uncertainties varied substantially across fitted components and depended strongly on spectral separation, as shown in Figure 7. These standard errors are estimated from the local curvature of the nonlinear least-squares solution and therefore indicate how well each fitted parameter is constrained by the available spectral information. In both data sets, the broad, overlapping lower-energy components showed substantially greater uncertainty than the more isolated high-energy components. This effect was most pronounced for the individual line width terms, σ and γ, whereas peak-center and total Voigt fwhm were generally better constrained.

**Figure 7:**
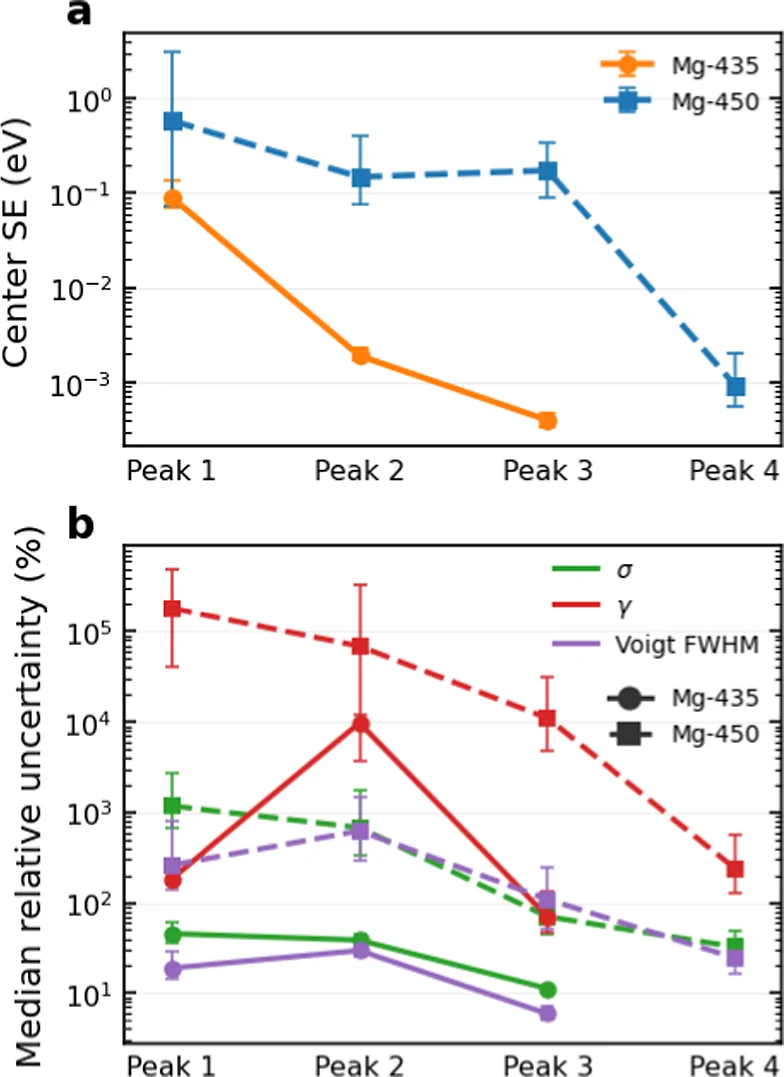
Covariance-derived fitting uncertainty across the Mg-435 and Mg-450 spectral maps. (a) Median peak-center standard error by component. (b) Median relative uncertainty in σ, γ, and total Voigt fwhm. Error bars indicate the interquartile range.

The uncertainty distributions were consistent with the behavior observed in the synthetic validation. In particular, the broad, overlapping lower-energy components exhibited the greatest parameter uncertainty, reinforcing the synthetic result that spectral overlap limits unique recovery of individual line-shape terms. γ showed especially large relative uncertainty for several of these components because neighboring peaks obscure the profile wings, allowing changes in γ to be partially compensated by changes in σ with only a small effect on the total fitted line shape. As has previously been shown for Gaussian/Lorentzian curve fitting, this coupling can limit the uniqueness with which the individual line-shape contributions are resolved even when the overall spectral profile is reproduced accurately.^31,32^ Analysis across the full spectral maps further revealed that this overlap-dependent loss of identifiability was systematic across the data sets, a trend that would be difficult to establish from the small number of representative fits typically examined in spectral deconvolution. Accordingly, the fitted Voigt components are interpreted as effective spectral descriptors rather than unique microscopic transitions, and the individual σ and γ terms are treated primarily as relative indicators of line-shape character rather than precise measurements of independent broadening mechanisms.

The highest-energy component is better suited for line width interpretation because a larger portion of its line shape, including the high-energy flank, remains exposed from neighboring contributions. Its consistently lower uncertainty and recovery of a finite Lorentzian term provide stronger evidence of mixed Gaussian- and Lorentzian-like character, although covariance still limits precise quantitative separation. For the more strongly overlapping components, fitted center, integrated contribution, and total Voigt width are more robust descriptors than the exact σ–γ partition. Because the same preprocessing, initialization, constraints, and fitting procedure were applied across each map, spatial variation in these quantities can still be interpreted comparatively as systematic changes in spectral behavior.

Application to the Mg-450 data set reveals well-defined and reproducible structure in the fitted results (Figure 8). Peak center energies are clearly separated by index (Figure 8a), with mean values near 1.58, 1.72, 1.90, and 2.02 eV for peaks 1–4, respectively, and relatively narrow distributions. This separation indicates that each fitted peak consistently represents a distinct spectral contribution across the data set. The relationship between peak area and center energy (Figure 8b) further highlights clustering by peak index, with limited overlap between adjacent peaks. Peak 3 exhibits the highest concentration of larger-area contributions, indicating its dominant role in the overall emission, while peak 4 remains comparatively lower in intensity. Together, these trends demonstrate that the fitting procedure yields stable peak assignments and captures both the consistency and variability of fitted behavior across the sample.

**Figure 8:**
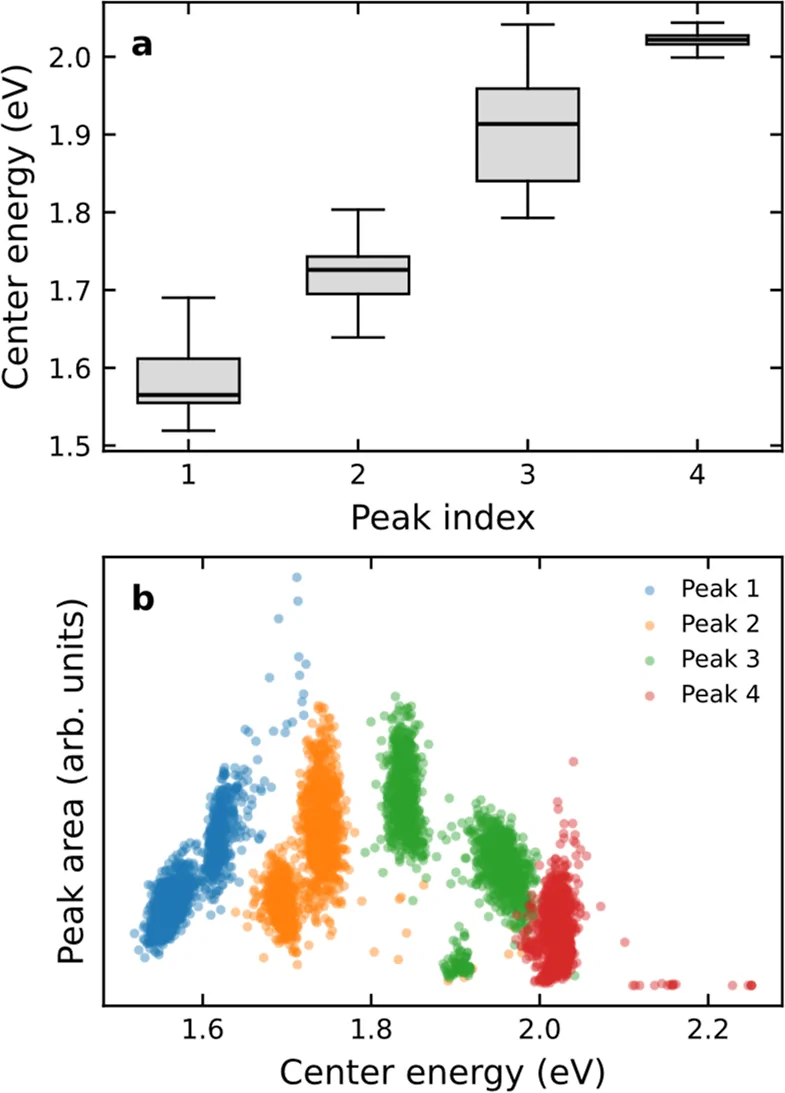
Statistical summary of Voigt fitting results across the full Mg-450 data set. (a) Boxplots of peak center energies grouped by peak index show clear separation of fitted spectral components. (b) Scatter plot of peak area (arb. units) versus center energy illustrates the distribution and relative contributions of each peak, with clustering reflecting variations in emission intensity across the data set.

Figure 9 compares the spatial distribution of the center energy of the dominant peak for Mg-435 (Figure 9a) and Mg-450 (Figure 9b), with corresponding peak-energy histograms shown below. Mg-435 exhibits a largely uniform map with minimal spatial variation, indicating a relatively homogeneous emission landscape. In contrast, Mg-450 displays pronounced spatial heterogeneity, with distinct regions of higher and lower emission energy. Similar spatial mapping approaches have been used to resolve local bandgap variations in polycrystalline absorbers.^33^ Because only one film was analyzed for each growth condition, the observed differences are interpreted as differences between these two representative films rather than as statistically established effects of Mg sublimation temperature.

**Figure 9:**
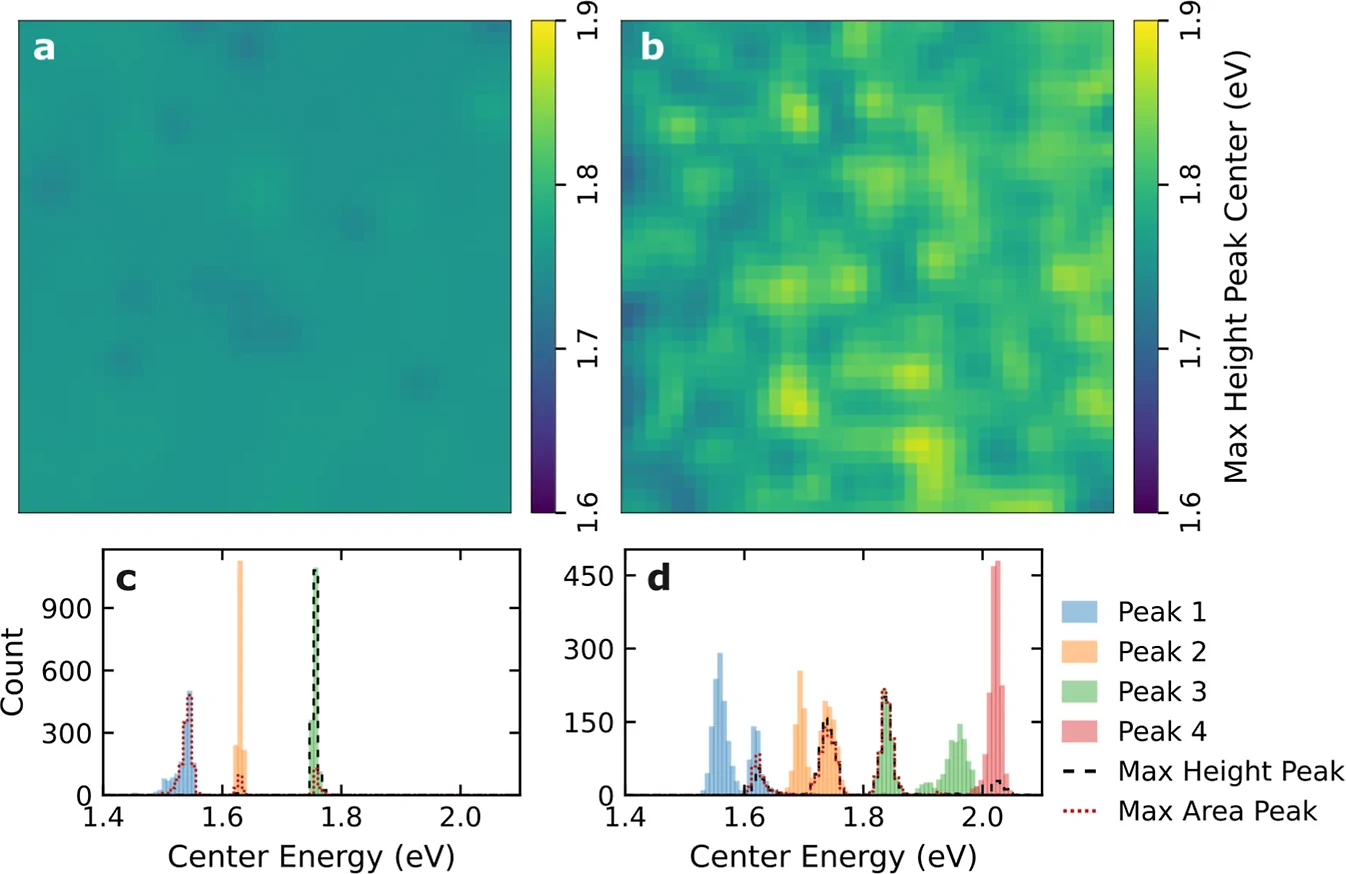
Comparison of spatial maps of the center energy (eV) corresponding to the highest peak height for samples (a) Mg-435 and (b) Mg-450 using the same energy scale. Sample Mg-435 exhibits a relatively uniform distribution, while sample Mg-450 shows increased heterogeneity with distinct regions of higher and lower emission energy. The color scale is consistent between maps to enable direct comparison. Histogram of peak center energies by index (color-coded) for (c) Mg-435 and (d) Mg-450, with overlays highlighting maximum peak area (red dotted) and highest-intensity peaks (black dashed).

The histograms (Figure 9c,d) further highlight differences in spectral behavior between the two samples. Mg-435 is dominated by a single primary emission feature, with most of the peak area concentrated in peak 1, while the highest-intensity contributions are consistently associated with peak 3. In contrast, Mg-450 exhibits multiple well-separated peak populations, with peak 3 (≈1.90 eV) most frequently contributing to the overall emission, while peaks 1 and 2 also appear as the highest-area or highest-intensity features in many spectra. These trends indicate that Mg-435 is characterized by a simpler and more uniform emission structure, whereas Mg-450 shows increased spectral complexity and spatial variability. Together, these results demonstrate that the fitting framework captures both the consistency of homogeneous samples and the structured heterogeneity present in more complex systems. To verify that the observed difference in spectral complexity was not an artifact of the selected model order, both data sets were reanalyzed using three- and four-component fits; Figure S4 shows that Mg-450 retains substantially greater spatial heterogeneity than Mg-435 under either model order.

The combination of spatial heatmaps and population-level statistics provides a compact, data-driven “fingerprint” of each film that captures both local and global characteristics of the emission behavior. Heatmaps preserve spatial structure, revealing whether variations occur as coherent domains or random fluctuations, while accompanying distributions (e.g., histograms of peak energy, width, and intensity) quantify the overall spread and relative prevalence of these features across the data set. Together, these representations encode not only the central tendencies of the material response but also the variability and spatial organization of that response. Because this fingerprint is derived from quantitative spectral descriptors extracted through a consistent analysis framework, it enables direct comparison between films and across processing conditions, allowing subtle but systematic differences in material behavior to be distinguished from baseline variability inherent to the measurement and fitting process.^23^

The Voigt decomposition provides a quantitative description of the fitted Gaussian and Lorentzian line width terms across the resolved spectral components. As shown in the σ versus center energy plot for Mg-450 (Figure 10a), peak 1 exhibits the largest σ values, corresponding to the broadest features. The first peak consistently approaches the maximum allowed σ value, reflecting its comparatively broader character and potential sensitivity to imposed fitting bounds. This contribution decreases systematically with increasing peak index. However, the box plots of γ by peak index (Figure 10b) show that γ is near the lower end of the fitted range for peaks 1–3, indicating that the fitted Lorentzian contribution is small for these components but not necessarily that Lorentzian-type broadening is physically absent. As shown by the uncertainty analysis, these overlapping peaks are also among the least well constrained, limiting independent interpretation of γ. In contrast, peak 4 exhibits both larger and more variable γ values, consistent with its greater spectral isolation and improved recovery of the Lorentzian line width term.

**Figure 10:**
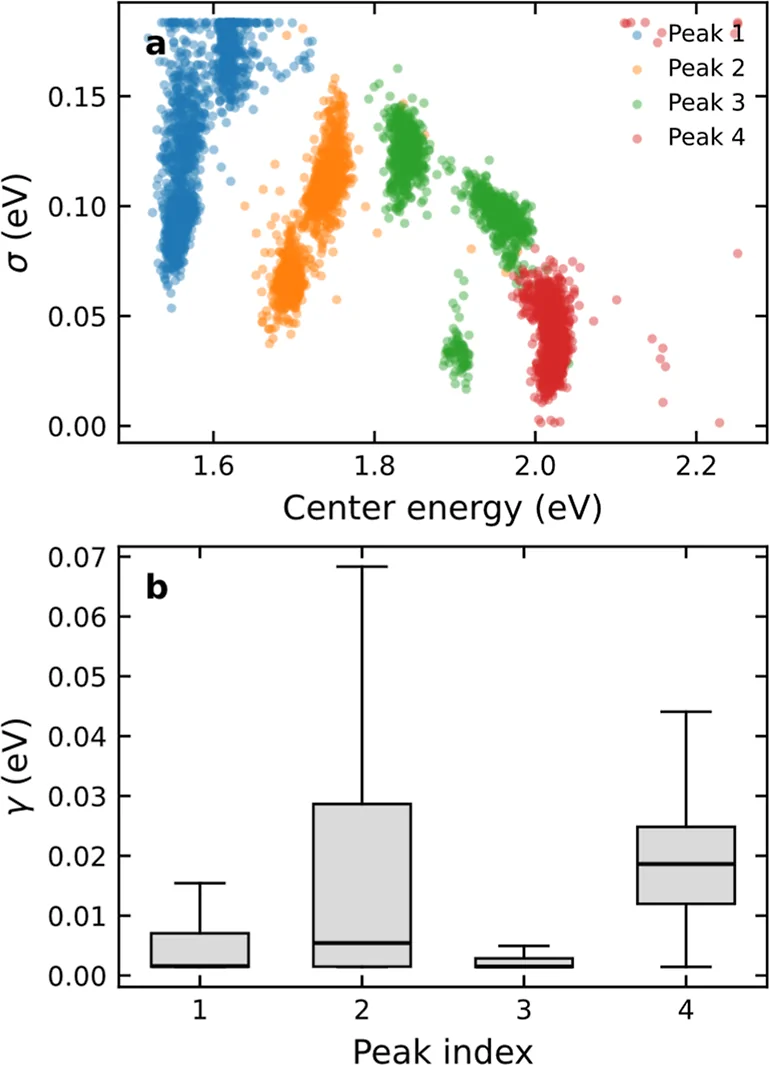
(a) Scatter plot of Gaussian width (σ) versus peak center energy, showing that peak 4 has the lowest Gaussian width among the fitted peak groups. (b) Boxplots of Lorentzian width (γ) for all four peaks, highlighting the increased magnitude and variability of the fitted Lorentzian line width in peak 4.

This distinction motivates the use of peak 4 for evaluating the σ/γ ratio in Figure 11a. Because peaks 1–3 have small and poorly constrained γ values, the ratio is not reliably interpretable for those peaks. For peak 4, however, the σ/γ ratio captures meaningful variation in the balance between fitted Gaussian and Lorentzian contributions. Spatial mapping of this ratio reveals regions of systematic differences in fitted line-shape character, providing a spatial descriptor of local peak-shape variation. Higher σ/γ values indicate a greater fitted Gaussian contribution, which can be associated with inhomogeneous broadening processes such as compositional variation, strain disorder, or unresolved spectral contributions, but should not be uniquely assigned to any one mechanism. In contrast, lower σ/γ values indicate relatively greater fitted Lorentzian character and may be consistent with increased homogeneous broadening contributions, including lifetime-related or exciton–phonon effects. These assignments remain qualitative because covariance between σ and γ limits precise separation of the two contributions. This distinction is useful in systems with multiple broadening mechanisms, such as hybrid perovskites.^34^

**Figure 11:**
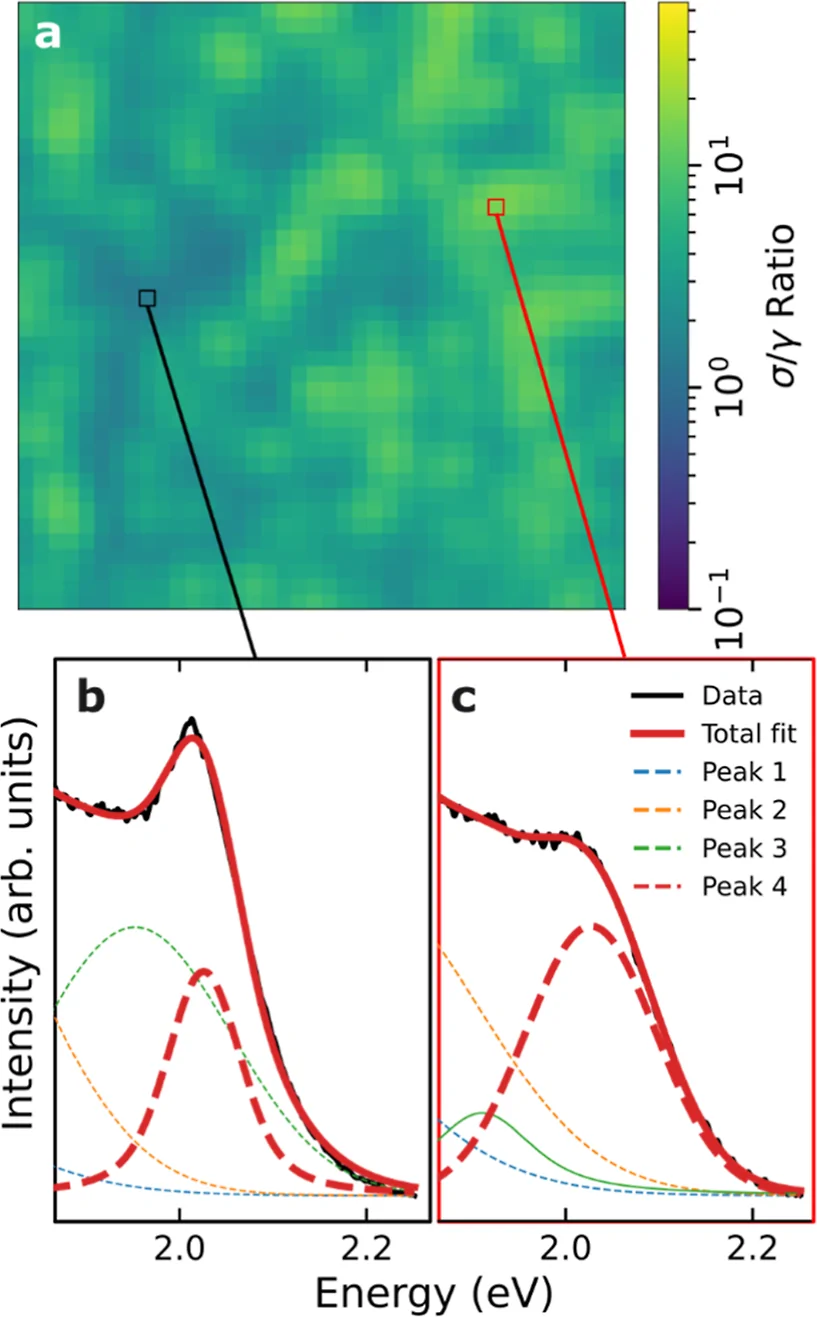
(a) Spatial map of the σ/γ ratio for peak 4 in Mg-450, highlighting variation in the relative fitted Gaussian to Lorentzian contribution. Representative Voigt fits showing spectra with (b) higher and (c) lower Lorentzian character (corresponding to lower and higher σ/γ ratios, respectively), with peak 4 indicated by the bold dashed red line.

To further illustrate these trends, representative Voigt deconvolutions for spectra from pixels with low (Figure 11b) and high (Figure 11c) σ/γ ratios, respectively, are provided. The spectrum with a high σ/γ ratio exhibits broader features, whereas the low σ/γ spectrum is characterized by narrower peaks with more Lorentzian character. These results underscore the utility of tracking spatial variation in fitted line-shape behavior and provide a framework for correlating peak shape parameters with structural or electronic variations across the sample.

## 4 Conclusion

This work presents a high-throughput Python-based framework for automated Voigt deconvolution of luminescence spectral maps. By combining curvature-informed peak initialization, structured line width optimization, constrained model-order selection, and nonlinear least-squares fitting, the workflow enables consistent extraction of peak-resolved parameters from large cathodoluminescence data sets. The approach addresses a key limitation in spectral map analysis: the need to resolve overlapping emission bands across thousands of spectra without relying on manual fitting or spectrum-by-spectrum intervention.

Application to CdMgSeTe thin films demonstrated that the fitted Voigt parameters provide quantitative descriptors of both spectral structure and spatial heterogeneity. The synthetic validation and covariance analysis further showed that peak center and total Voigt line width are generally more robustly recovered than the individual Gaussian and Lorentzian line width contributions, particularly for strongly overlapping components. Despite these identifiability limitations, consistent application of the fitting framework enables reproducible comparison of spatial and population-level spectral trends across samples.

Overall, the framework provides a reproducible and scalable method for converting hyperspectral luminescence maps into quantitative peak-resolved spectral descriptors. Together, the peak-resolved statistics, spatial heatmaps, and line width descriptors form a quantitative spectral fingerprint that captures both local variation and population-level emission behavior across each film. This fingerprint supports more consistent comparison between samples and processing conditions and provides a practical analysis pathway for complex semiconductor materials where local variations in composition, disorder, strain, or recombination behavior influence the measured spectra. Future work will extend the workflow to additional spectroscopic techniques, including Raman spectroscopy, where Voigt-based deconvolution is commonly used to analyze overlapping spectral features.

## Supplementary Material



## Notes

SEM image of analyzed thin film; pseudocode and implementation details for fixed-order Voigt fitting; curvature-based weighting function; model-order selection analysis; synthetic validation results; alternative model-order spatial maps (PDF)
