# Supplementary material for: High-Throughput Voigt Deconvolution of Luminescence Spectral Maps with Curvature-Informed Peak Initialization
Source: ACS Omega. 2026 Oct 21;11(43):67136–46. doi: 10.1021/acsomega.6c07689 (PMC13737981; doi:10.1021/acsomega.6c07689)
Supplement: Supplementary file 1 [file ao6c07689_si_001.pdf]

High-Throughput Voigt Deconvolution of Luminescence Spectral Maps with Curvature-Informed Peak Initialization

Melissa A. Wunch; † Luna S. Garcia; † Jordan Barry; † Chris Thron; † Taylor B. Harvey†,*

†Department of Science and Mathematics, Texas A&M University-Central Texas, Killeen, TX 76549, USA

* Corresponding Author: tharvey@tamuct.edu

Supporting Information

S1. Experimental

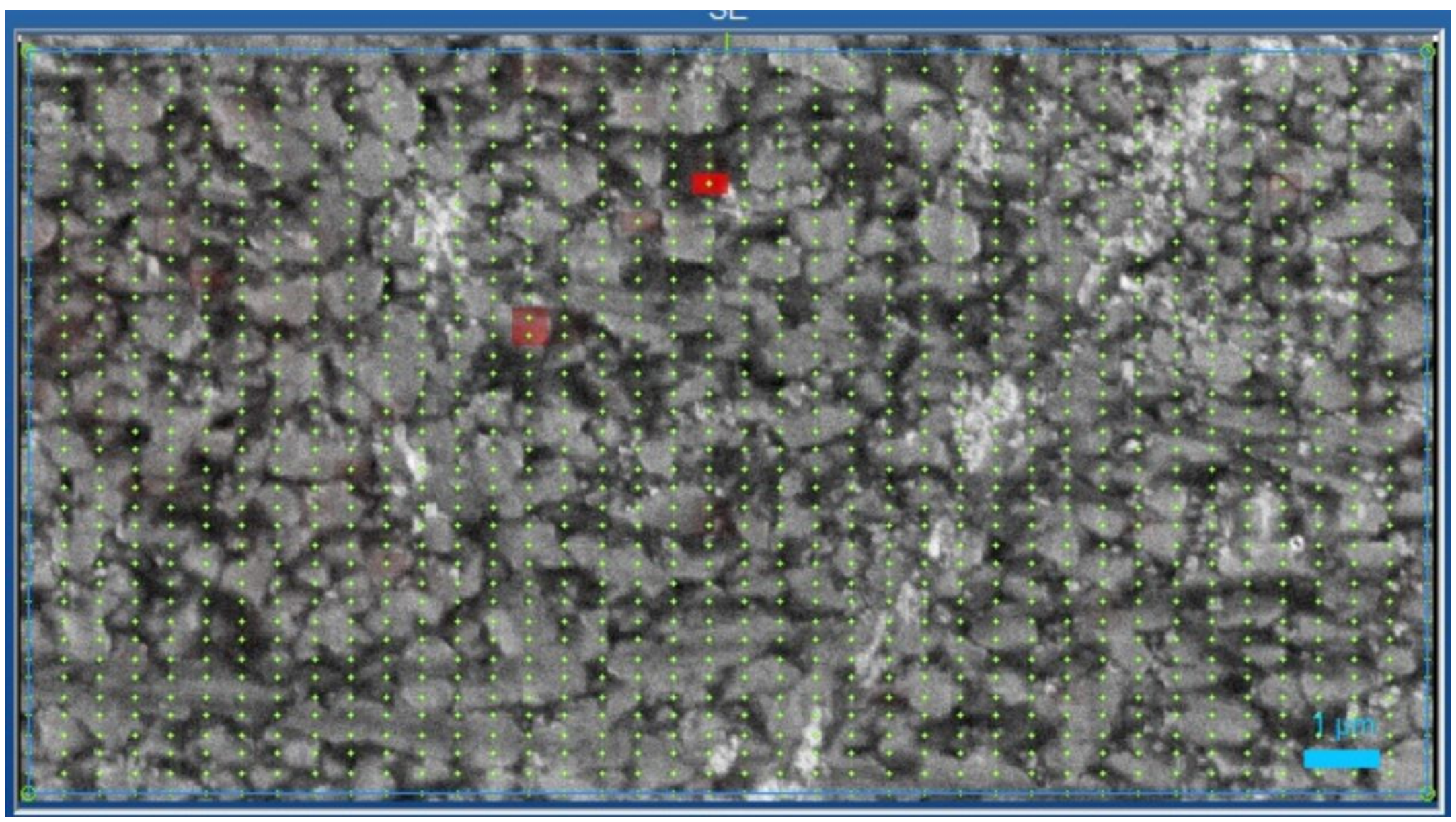

Figure S1. SEM image of the thin film used to collect the mapping data processed by the Python algorithm

Pseudocode for Fixed-Order Voigt Fitting Workflow

The fitting workflow was implemented as a curvature-initialized, constrained nonlinear least-squares procedure, summarized in Algorithm 1. Each spectrum was converted to an energy basis with the appropriate Jacobian correction, smoothed for robust peak-center initialization, and then fit using a fixed-order sum of Voigt components with bounded parameters and optional curvature-based weighting.

Algorithm

Inputs

Raw spectral map containing wavelength axis, λ, and one or more spectra, I(λ)
Fixed number of Voigt components, n, selected from the dataset-wide candidate-count analysis
Fourier smoothing parameters: 50 endpoint points and 10 retained Fourier components
Gaussian smoothing parameter: σ = 2 grid points
Parameter bounds for amplitude, center position, Gaussian linewidth, σ, and Lorentzian linewidth, γ
Curvature-weighting factor, α = 3
A 5 × 5 logarithmically spaced (σ_0, γ_0) initialization grid

Outputs
For each fitted spectrum:
Amplitude, center energy, σ, and γ for each Voigt component
Total Voigt full width at half maximum (FWHM)
Peak height and integrated peak area
Covariance-derived parameter standard errors and 95% confidence intervals
σ–γ parameter correlation
Residual sum of squares (RSS)
Root-mean-square error (RMSE)
Normalized root-mean-square error (NRMSE)
Coefficient of determination, R^2
Convergence status and diagnostic information

Procedure
1. Load spectral dataset
1.1 Read the spectral map from the input file.
1.2 Assign the first column as the spectral axis and all remaining columns as individual spectra.

2. For each spectrum in the selected dataset
2.1 Convert the spectrum to the fitting domain
If fitting in energy space:
E = 1239.841984 / λ
Apply the Jacobian intensity correction:
$I_E = I_\lambda \times \lambda^2 / 1239.841984$
Sort the resulting data in order of increasing energy.
2.2 Interpolate onto a uniform energy grid
Interpolate the converted spectrum onto a uniformly spaced energy grid containing the same number of points as the original spectrum.
2.3 Generate smoothed spectra
Generate a Fourier-smoothed spectrum using endpoint-conditioned truncated Fourier reconstruction with 50 endpoint points and 10 retained Fourier components.

Generate a Gaussian-smoothed spectrum using $\sigma = 2$ grid points.
Use the Fourier-smoothed spectrum for curvature-based candidate identification and the Gaussian-smoothed spectrum for nonlinear fitting.

2.4 Apply baseline and intensity corrections

No additional baseline subtraction is applied when instrument-level baseline correction has already been performed.
Shift the fitting spectrum by subtracting its minimum value so that the minimum fitting intensity is zero.

2.5 Identify initial peak-center candidates

Compute the discrete second derivative of the Fourier-smoothed spectrum.
Identify local minima in the second derivative and exclude candidates within 3% of either spectral edge.
Rank remaining candidates by curvature magnitude, with the most negative second-derivative minima ranked first.
Retain up to n candidate centers.
If fewer than n candidates are available, generate fallback centers distributed across the fitted energy range with 5% edge padding while maintaining approximately 3% of the energy span as a minimum separation from existing centers.
Sort all initial centers in ascending energy.

2.6 Initialize Voigt parameters and fitting bounds

Estimate each initial amplitude as 60% of the local fitted intensity at the corresponding center.
For a fitted energy span ΔE_span and energy-grid spacing ΔE:
Amplitude ≥ 0
Each center may vary within ±10% of ΔE_span from its initialized position, subject to the acquired spectral range.
$\sigma\_min = 2\Delta E$
$\gamma\_min = 2\Delta E$
The broadest low-energy component is permitted a σ upper bound of 25% of the fitted energy span; the remaining components use the configured linewidth upper bound.
γ_max = 12.5% of the fitted energy span.
Initialize nominal linewidth values at 5% of the fitted energy span for σ and 2.5% for γ.

2.7 Construct curvature-based fitting weights

Compute the discrete second derivative of the spectrum used for fitting. Full details given separately below.

2.8 Optimize initial linewidth guesses

Evaluate a 5 × 5 logarithmically spaced grid of representative initial linewidth values.
The σ_0 starting values correspond approximately to:
0.0199, 0.0315, 0.0500, 0.0792, and 0.1256 of the fitted energy span.
The γ_0 starting values correspond approximately to:
0.0100, 0.0158, 0.0250, 0.0396, and 0.0628 of the fitted energy span.

For each (σ_0, γ_0) combination:
Construct the full initial parameter vector.
Perform bounded nonlinear least-squares fitting.
Calculate the resulting sum of squared errors.
Select the initialization combination yielding the lowest SSE and use its linewidth values to initialize the final fit.
The initialization grid is used only to improve convergence. The final fitted linewidths remain free to vary throughout their full allowed bounds.

2.9 Perform the final constrained Voigt fit

Model the spectrum as the sum of n Voigt components evaluated using the Faddeeva formulation.
Simultaneously optimize amplitude, center, σ, and γ for all components using bounded nonlinear least-squares fitting.
Apply the curvature-based relative weights during optimization.
Sort fitted peaks by increasing center energy.
Calculate the summed fitted spectrum and residual vector.

2.10 Calculate fitted peak quantities and uncertainty

For each fitted component:
Store amplitude, center energy, σ, and γ.
Calculate peak height numerically.
Integrate peak area numerically.
Calculate total Voigt FWHM from the fitted linewidth terms.
Calculate covariance-derived standard errors.
Calculate approximate 95% confidence intervals.
Calculate the σ–γ parameter correlation.
Record parameter proximity to imposed bounds.
For the complete spectrum:
Calculate RSS.
Calculate RMSE.
Calculate NRMSE.
Calculate R^2.
Record residual statistics and covariance diagnostics.

2.11 Handle failed fits

If the nonlinear fit fails:
Record the spectrum index.
Record the fitting error.
Mark the fit as unsuccessful.
Continue processing the next spectrum.

3. Save results

Export peak-level and spectrum-level fitting results to structured output files for downstream statistical analysis and spatial mapping.

Note:

Voigt profiles were evaluated using the Faddeeva function implemented in SciPy, and constrained nonlinear least-squares optimization was performed using scipy.optimize.curve_fit. Production analyses used the numerical preprocessing, parameter bounds, weighting function, and linewidth-initialization settings reported above.

Curvature-Based Weighting Function

To increase the influence of spectral regions containing stronger local structure, the nonlinear least-squares fit used a curvature-based relative weighting scheme. For each spectrum, the discrete second derivative was calculated from the data used for fitting, and the pointwise weight was defined as

$$w_i = 1 + \alpha \frac{|S_i''|}{P_{95}(|S''|)} \quad (S1)$$

where S_i'' is the local second derivative, $P_{95}(|S''|)$ is the 95th percentile of the absolute curvature magnitude for that spectrum, and $\alpha=3$ is the weighting factor. The two endpoint weights were retained at unity. Larger weights therefore increase the contribution of high-curvature regions to the fitting objective. The weighting was implemented in SciPy curve_fit through its sigma argument using $1/\sqrt{w_i}$. These values are numerical fitting weights and do not represent experimental measurement uncertainties.

S2. Results

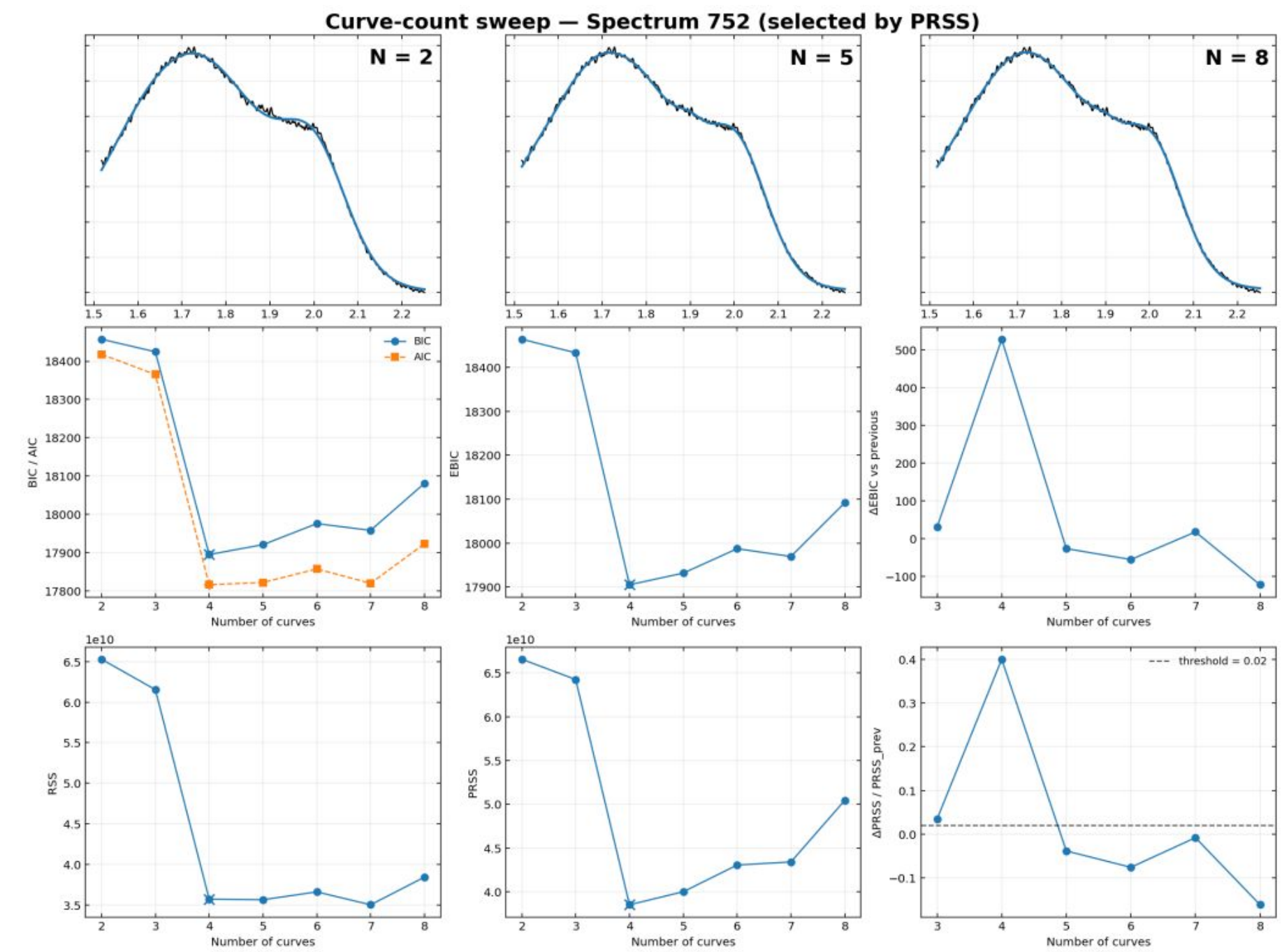

Figure S2. Curve-count (model order) sweep for a representative spectrum (Spectrum 752), illustrating the selection of the number of Voigt components. Top row shows example fits for n = 2, 5, and 8 components. Middle row presents model selection metrics, including Bayesian Information Criterion (BIC), Akaike Information Criterion (AIC), and the change in BIC (ΔBIC). Bottom row shows residual-based metrics, including residual sum of squares (RSS), penalized RSS (PRSS), and the change in PRSS (ΔPRSS). The PRSS criterion identifies n = 4 as the optimal model order, balancing goodness of fit and model complexity.

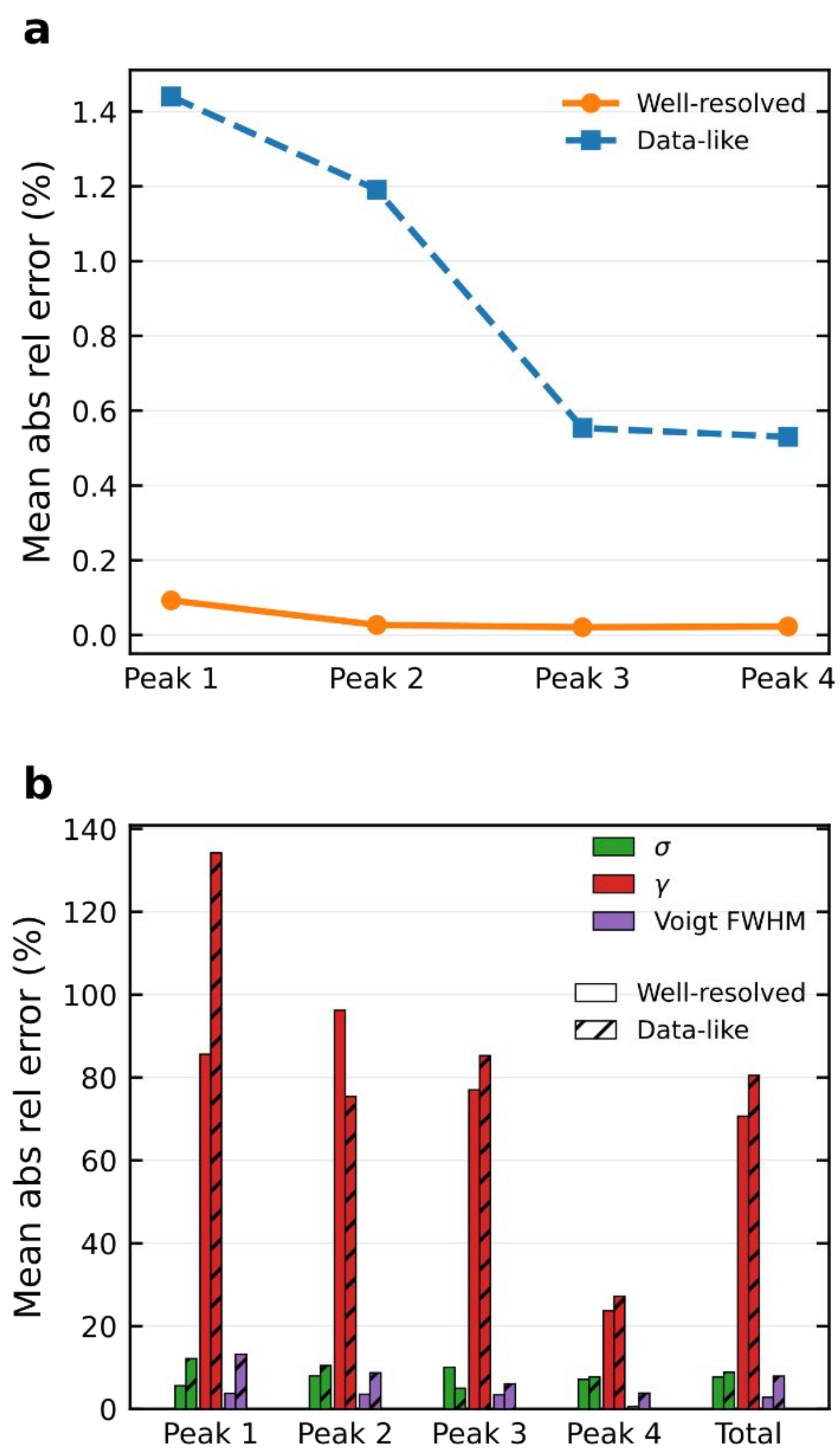

Figure S3. (a) Mean absolute error in recovered peak-center energy for the well-resolved and data-like cases. (b) Mean absolute relative error in fitted σ, γ, and total Voigt FWHM for each synthetic peak. Errors increase for overlapping components, particularly for γ, while peak-center and total-width recovery remain more robust.

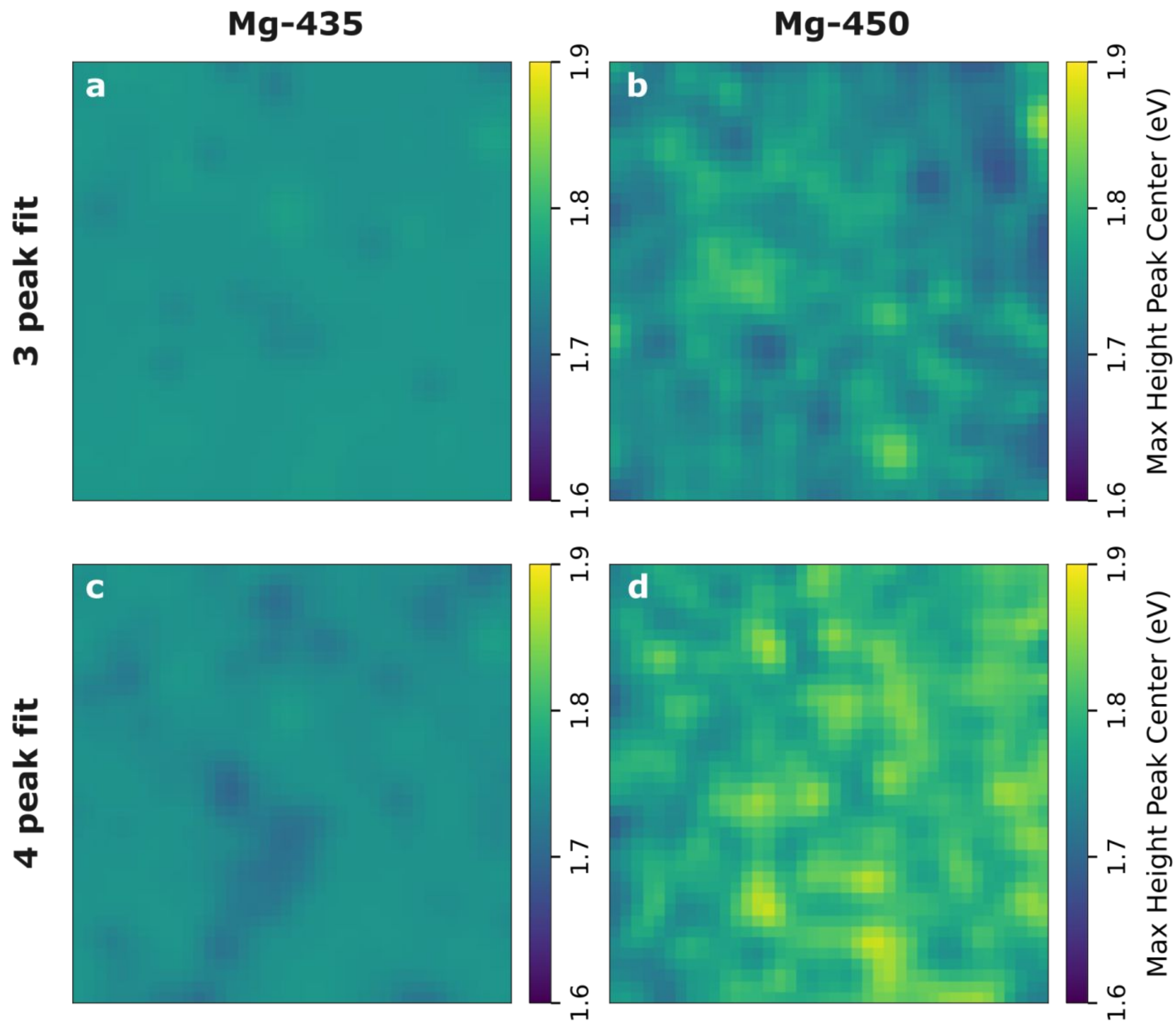

Figure S4. Spatial maps of the center energy corresponding to the highest-intensity fitted peak for Mg-435 and Mg-450 using common three-component fits (a,b) and four-component fits (c,d). Mg-450 (b,d) retains substantially greater spatial variation than Mg-435 (a,c) under both model orders, indicating that the observed difference in spectral heterogeneity is not an artifact of the selected number of Voigt components.
